# Risk-adjusted zero-inflated Poisson CUSUM charts for monitoring influenza surveillance data

**DOI:** 10.1186/s12911-021-01443-8

**Published:** 2021-07-30

**Authors:** Yueying Tan, Xin Lai, Jiayin Wang, Xuanping Zhang, Xiaoyan Zhu, Ka-chun Chong, Paul K. S. Chan, Jing Tang

**Affiliations:** 1 School of Computer Science and Technology, Xi’an Jiaotong University, Xi’an, 710049 China; 2 School of Public Health and Primary Care, The Chinese University of Hong Kong, Hong Kong, China; 3 Department of Microbiology, Faculty of Medicine, The Chinese University of Hong Kong, Hong Kong, China; 4 Department of Gynecology and Obstetrics, Luzhou People’s Hospital, Luzhou, China

**Keywords:** Cumulative sum control chart, Average run length, Zero-inflated Poisson regression, Risk adjustment, Influenza surveillance data

## Abstract

**Background:**

The influenza surveillance has been received much attention in public health area. For the cases with excessive zeroes, the zero-inflated Poisson process is widely used. However, the traditional control charts based on zero-inflated Poisson model, ignore the association between influenza cases and risk factors, and thus may lead to unexpected mistakes when implementing monitoring charts.

**Method:**

In this paper, we proposed risk-adjusted zero-inflated Poisson cumulative sum control charts, in which the risk factors were put to adjust the risk of influenza and the adjustment was made by zero-inflated Poisson regression. We respectively proposed the control chart monitoring the parameters individually and simultaneously.

**Results:**

The performance of our proposed risk-adjusted zero-inflated Poisson cumulative sum control chart was evaluated and compared with the unadjusted standard cumulative sum control charts in simulation studies. The results show that for different distribution of impact factors and different coefficients, the risk-adjusted cumulative sum charts can generate much less false alarm than the standard ones. Finally, the influenza surveillance data from Hong Kong is used to illustrate the application of the proposed chart.

**Conclusions:**

Our results suggest that the adjusted cumulative sum control chart we proposed is more accurate and credible than the unadjusted standard control charts because of the lower false alarm rate of the adjusted ones. Even the unadjusted control charts may signal a little faster than the adjusted ones, the alarm they raise may have low credibility since they also raise alarm frequently even the processes are in control. Thus we suggest using the risk-adjusted cumulative sum control charts to monitor the influenza surveillance data to alert accurately, credibly and relatively quickly.

## Background

Control chart is an important tool of the statistical process control (SPC), they are widely used in manufacturing industry to monitor the production process, in the past decades, they are also used in monitoring the public health data, such as the surgical performance monitoring [1] and influenza epidemics monitoring and predicting [2–4]. However, the researches before did not consider distribution of the influenza data.

Traditional control chart are used to monitoring the count data which follows the Poisson distribution or binomial distribution. But for influenza surveillance data from one hospital, the data may have excess zeroes and lower mean value. When monitoring this kind of data, the use of the Poisson model may underestimate the dispersion of the data and cause the control limits so tight that leads to higher false alarm rate. Thus for these processes, the zero-inflated Poisson (ZIP) distribution is used. Xie and Goh [5] first developed a ZIP model to monitor a near zero-defect process, in which the non-conformities caused by random shocks follows the Poisson distribution with parameter $\uplambda$, and the random shocks probability is *p*. In their research, a single Shewhart chart was proposed to monitor the ZIP processes.

Since then, several control charts for monitoring the ZIP process have been developed and studied. Xie et al. [6] provided some test methods to test the Poisson model against ZIP model to determine whether ZIP model should be used to monitor the process, and proposed a Shewhart control chart to monitor a process following the ZIP distribution. Sim and Lim [7] proposed a Shewhart *c*-chart, the upper control limit (UCL) of which is determined by the Jeffreys interval for the Poisson parameter $\uplambda$ and a Shewhart *np*-chart, which is constructed based on the Jeffreys prior interval or the Blyth-Still interval. And they used the two of two control rule to improve the performance. Chen et al. [8] developed a generalized ZIP (GZIP) model where there are multiple random shocks occurring, the shocks may have different probabilities and may lead to different Poisson distributions. For the GZIP model they proposed the Shewhart chart, cumulative sum (CUSUM) chart and ranked probability control charts to monitor the GZIP process and evaluated their performance, they are the first to applicate CUSUM control chart to monitor the ZIP processes.

Motivated by the GZIP control charts proposed by Chen et al., He et al. [9]. developed CUSUM chart to monitor the ZIP processes. They gave a clear definition of the *p*-CUSUM for monitoring the shift of the parameter *p* and $\uplambda$-CUSUM for monitoring the shift of the parameter $\uplambda$ individually, and proposed the *p*-$\uplambda$ CUSUM, combination of the *p*-CUSUM and $\uplambda$-CUSUM, when either of the two parameters shifts the chart will signal. They also proposed the *t*-CUSUM to monitor the two parameters simultaneously. From their comparison study, these different kinds of CUSUM control chart can be applied in different application scenarios. Fatahi et al. [10] proposed an exponentially weighted moving average (EWMA) control chart to monitor the ZIP process, and the EWMA control chart they proposed was proved to have relatively better performance than the previously developed charts. The ZIP-EWMA can be used to monitor the rare health-related events. The application of the ZIP-EWMA to needle-stick occurrence can show the high precision of the proposed chart. He et al. [11] proposed a new CUSUM control chart called CRL-ZTP CUSUM, which is the combination of a conforming run length (CRL) CUSUM chart and a zero truncated Poisson (ZTP) CUSUM chart. They also compared the performance of the CRL-ZTP CUSUM chart with the other ZIP CUSUM control charts and found that it is more effective when only the parameter *p* shifts and *t*-CUSUM is still the best control chart to defect the simultaneous shift of parameters *p* and $\uplambda$.

Recently, numbers of new control charts are used to better monitor the ZIP processes. Aly et al. [12] developed an adaptive exponentially weighted moving average (AEWMA) control chart for monitoring ZIP processes. They used the relative mean index (RMI) metric to compare the performance of the control charts and the result showed that the AEWMA has superior performance when the shift of the parameter is large. Alevizakos et al. [13]. proposed a generally weighted moving average (GWMA) control chart to monitor the ZIP process and they studied the ZIP-GWMA with different parameters setting and compared these charts with other charts studied before and the proposed chart can perform best under condition of shifts in both parameters. Alevizakos et al. [14] developed a double EWMA (DEWMA) chart with an upper time-varying control limit to monitor ZIP process and studied its performance by comparing with the other control charts, results showed that it is very effective especially in detecting shifts of *p* only and both the parameters simultaneously.

However, in the standard ZIP control chart, it is assumed that the probability of random shocks *p* and the mean value of Poisson distribution $\uplambda$ among the entire process are constants, which is unreasonable, especially when monitoring the health data such as the influenza data we consider in this paper. In practice, the occurrence of the health event is irregular, the parameters vary from day to day because of the variance of the impact factors such as temperature, humid, pressure and so on. Several researches have been done to demonstrate the association between the climatological parameters and the influenza activities, especially temperature, humidity and rainfall. Soebiyanto et al. [15]. studied the climatic factors on the epidemiology of influenza in two warm climate regions including Hong Kong, using the time series model, the results showed that Land Surface Temperature (LST), rainfall and relative humidity are the most important factors in the model. Researches [16–19] about the association between climate factors and the seasonal influenza activities among regions with temperate, subtropical and tropical climate showed the different impacts of the factors on different regions, suggesting the importance to include the climate factors when model the influenza activities. Lofgren et al. [20]. found that the seasonal influenza is not only effected by environment causes but also social causes. Besides, all the researches show the influenza activities have obvious seasonality. That’s why we need to adjust the CUSUM to relate the impact factors with the ZIP parameters instead of just considering them as constants.

There have been many studies on risk-adjusted control chart. Steiner et al. [1] proposed a risk-adjusted CUSUM chart to monitor surgical performance, the surgical risk of each patient is variant, the risk-adjusted CUSUM chart they proposed can reduce the false alarm rate as well as detect the unusual changes quickly. Grigg and Farewell [21] proposed a risk-adjusted sets method and made an overview of several risk-adjusted control charts and compared the performance of them. They found that the set method can detect the small changes quickly but along with the high false alarm rate, by contrast the CUSUM chart may raise less false alarm and can detect changes quickly. Liu et al. [22] proposed a new risk-adjusted EWMA chart based on score test and can simultaneously monitor location and scale parameters, the comparison with the risk-adjusted CUSUM showed that the proposed charts performed better.

Several studies about influenza surveillance data monitoring have been done in the past decades. Cowling et al. [2]. proposed a time-series method to monitor the short-term influenza data, which is a dynamic linear model. They compared the time-series method, regression and CUSUM chart on the Hong Kong and US data to predict the peak season of influenza, the results show that the time-series method they proposed has the best performance on Hong Kong data and the time-series method and CUSUM have similar performance on US data. The CUSUM they compared with is a simple model based on the recent 7 weeks data. Boyle et al. [3] used CUSUM, forecast based on historical data and prediction based on Google searching data to predict the influenza epidemics, the comparison in their paper showed that the combination of the CUSUM and forecasting method. Zhang et al. [4] used seasonal autoregressive integrated moving average (SARIMA) model with Google trends and temperature and regression tree analysis to predict the seasonal influenza breakouts.

In this paper, we propose a risk-adjusted ZIP CUSUM control chart to monitor the ZIP process in which the parameters are affected by the impact factors. Seasonality is also considered in our chart using Fourier series type approach. We designed the risk-adjusted *p*-CUSUM to detect the shift of parameter *p* while $\uplambda$ is in control, the risk-adjusted $\uplambda$-CUSUM to detect the shift of parameter $\uplambda$ when *p* is in control and the risk-adjusted *t*-CUSUM to detect the shift of both the parameters. We use the ZIP regression to make the adjustment, as Lambert [23] did in 1992. The regression can be made using Phase I data, the coefficients obtained from the regression will be used to generate variant parameters. Taking the impact factors into consideration, the adjusted CUSUM chart can coincide with the real conditions better, especially for the health data. The proposed chart can raise less false alarm, ensure the credibility and accuracy of the signals. We make a simulation study to demonstrate the stability of our proposed chart under the in-control cases and apply the chart to the Hong Kong influenza surveillance data, the results also show the good performance of the chart.

## Model and method

### Zero-inflated Poisson model

The zero-inflated Poisson (ZIP) model is one of the generalization of the standard Poisson model, based on a distribution allowing excess zero observations. Under a ZIP model, it is assumed that there are some random shocks causing the non-conformities and it follows a Poisson distribution with parameter $\uplambda$, while the probability of the occurrence of the random shocks is *p*. As defined by Xie and Goh [5], *X* is the observation following ZIP distribution, the probability density function (pdf) of *X* can be defined as follows:

1 $$\begin{aligned} f(X=x ; p, \uplambda )=\left\{ \begin{array}{l} 1-p+p e^{-\uplambda }, \quad x=0 \\ p \frac{\uplambda ^{x} e^{-\uplambda }}{x !}, \quad x>0 \end{array}\right. \end{aligned}$$

In practice, the parameters *p* and $\uplambda$ are usually unknown, they can be estimated using the observed numbers of non-conformities $X_1,X_2,\ldots ,X_n$ by the maximum likelihood estimation method [24]:

2 $$\begin{aligned} \begin{aligned} \hat{\uplambda }&=\frac{\bar{X}}{\hat{p}} \frac{n-n_{0}}{n} \\ \hat{p}&=\frac{1}{1-e^-\hat{\uplambda }} \frac{n-n_{0}}{n} \end{aligned} \end{aligned}$$

where $\hat{\uplambda }$ and $\hat{p}$ are the estimators, $\bar{X}$ is the mean of the positive observed $X_i$, *n* is the number of observations and $n_0$ is the number of observed zeros.

### Standard ZIP CUSUM

The cumulative sum (CUSUM) control chart was first proposed by Page [25] and this kind of control chart was initially proposed for monitoring industrial production processes, meanly contributing to detect the unusual process changes. The standard tabular CUSUM statistics is as follows:

3 $$\begin{aligned} C_{t}=\max \left( 0, C_{t-1}+W_{t}\right) , \quad t=1,2, \ldots \end{aligned}$$

where $C_t$ is the CUSUM statistics at time *t*, $C_0=0$ and $W_t$ is the score of the *t* th observation based on the log-likelihood ratio. We assume that the probability density function(pdf) of the distribution under null hypothesis is $f_0$ and $f_1$ under alternative hypothesis, thus the score $W_t$ can be log$\left( f_1/f_0 \right)$. In this paper we want to detect the increase of the number of influenza cases, so we just consider the upper shift of the parameters, namely we just consider the upper-sided CUSUM control chart in our work.

The CUSUM control chart will signal when $C_t>h$, *h* is the control limit determined to achieve the specified in-control performance. When the CUSUM statistics exceed the control limit, we say the process out of control, and the chart will raise alarm to the practitioner to handle the unexcepted changes timely. Chen et al. [8]. was the first to apply CUSUM charts to monitor the generalized ZIP process. He et al. [9] proposed *p*-CUSUM and $\uplambda$-CUSUM respectively to monitor the shift of *p* or $\uplambda$ only, and *t*-CUSUM to monitor the shift of the two parameters simultaneously. The CUSUM they proposed have superior performance against the Shewhart chart when detect small shifts. The CUSUM score $W_t$ of their standard ZIP-CUSUM can be obtained from the pdf of ZIP distribution. For *p*-CUSUM chart, $p_0$ and $p_1$ are respectively the random probabilities under null and alternate hypotheses, $p_1$ represents a specific shift of $p_0$. To detect the upper shift of the parameter, $p_1>p_0$. The score $W_t$ is given by:

4 $$\begin{aligned} W_{t}=\left\{ \begin{array}{l} \log \frac{1-p_{1}+p_{1} e^{-\uplambda _{0}}}{1-p_{0}+p_{0} e^{-\uplambda _{0}}}, \quad X_{t}=0 \\ \log \frac{p_{1}}{p_{0}}, \quad X_{t}>0 \end{array}\right. \end{aligned}$$

Combined with the Eq. (3), the CUSUM statistics can be calculated and monitor the processes where *p* shifts. Similarly, the score $W_t$ of $\uplambda$-CUSUM is:

5 $$\begin{aligned} W_{t}=\left\{ \begin{array}{l} \log \frac{1-p_{0}+p_{0} e^{-\uplambda _{1}}}{1-p_{0}+p_{0} e^{-\uplambda _{0}}}, \quad X_{t}=0 \\ X_{t} \log \frac{\uplambda _{1}}{\uplambda _{0}}+\left( \uplambda _{0}-\uplambda _{1}\right) , \quad X_{t}>0 \end{array}\right. \end{aligned}$$

And the score $W_t$ of *t*-CUSUM can be defined as:

6 $$\begin{aligned} W_{t}=\left\{ \begin{array}{l} \log \frac{1-p_{1}+p_{1} e^{-\uplambda _{1}}}{1-p_{0}+p_{0} e^{-\uplambda _{0}}}, \quad X_{t}=0 \\ X_{t} \log \frac{\uplambda _{1}}{\uplambda _{0}}+\left( \uplambda _{0}-\uplambda _{1}\right) +\log \frac{p_{1}}{p_{0}},\quad X_{t}>0 \end{array}\right. \end{aligned}$$

In the standard CUSUM control chart, the in-control parameters are designed to be constants. In simulation study, they are assumed to be already known, however in practice they need to be estimated from Phase I in-control data by MLE.

### Zero-inflated Poisson regression

In influenza surveillance data monitoring, the parameters of the ZIP processes are always affected by the real factors such as temperature, humid, rain or not etc. It is unreasonable to assume that the random shock probability *p* and the mean number of the flu cases per day $\uplambda$ are constant values. An adjustment for the parameters is therefore necessary to ensure they vary from day to day owing to the effect of the real impact factors. The ZIP regression by Lambert [23] can be used to do this adjustment. The coefficients of the two parameters *p* and $\uplambda$ can be estimated by maximum likelihood estimation(MLE) using the historical data. The parameters *p* and $\uplambda$ satisfy:

7 $$\begin{aligned} \begin{aligned} \log (\uplambda )&=\varvec{\alpha }\varvec{x}+c \\ \log i t (p)=\log \left( \frac{p}{1-p}\right)&=\varvec{\beta } \varvec{z}+k \end{aligned} \end{aligned}$$

where $\varvec{\alpha }$ and $\varvec{\beta }$ are coefficient vectors, $\varvec{x}$ and $\varvec{z}$ are the vectors of the impact factors, they can either be the same or not for *p* and $\uplambda$, *c* and *k* are intercept parameters. An offset term $\log (n)$ can also be considered here in the Poisson regression of $\uplambda$, *n* is the number of the total population of the monitored region. Thus the Eq. (7) can be transformed to:

8 $$\begin{aligned} \begin{aligned} \log (\uplambda )&=\varvec{\gamma }\varvec{x}+\log (n)+d \\ \log i t (p)=\log \left( \frac{p}{1-p}\right)&=\varvec{\beta } \varvec{z}+k \end{aligned} \end{aligned}$$

where $\log (n)$ is called the offset term of the ZIP regression, since $\uplambda$ is the daily number of cases in the monitored region, we set a variable $\mu =\uplambda /n$ to represent the prevalence rate of the region, thus we can monitor the prevalence rate directly, which can be more comparable and interpretable.

The parameters *p* and $\uplambda$ can be calculated when the coefficients and the impact factors are known. To estimate the coefficients and intercepts in application where they are unknown, we simply use the *zeroinfl()* function from package *pscl* in R to fit the ZIP data.

### Risk-adjusted ZIP CUSUM chart

To take the impact factors under consideration, we can adjust the CUSUM based on the factors. We define $p_t$ as the adjusted random shock probability, varying with the $\varvec{\beta }$ coefficient vector and the intercept *k* mentioned before, and $\uplambda _t$ varying with the $\varvec{\alpha }$ coefficient vector and the intercept *c*, the coefficients are obtained by ZIP regression from the in-control data. The values of $p_t$ and $\uplambda _t$ can be obtained by Eq. (7) using the current factor $\varvec{x}$ and $\varvec{z}$. For shock probability *p* we define the hypotheses $H_0$ and $H_1$ based on the odds ratio and for the mean number of flu cases $\uplambda$ we define the hypotheses based on the relative risk. Let ${OR}_0$ and ${OR}_1$ represent the odds ratios under null and alternate hypotheses. The estimated risk of shock equals to $p_t$, then the odds of shock equals to $p_t/(1-p_t)$. Under $H_0$ the odds of shock for *t* th observation is ${OR}_0p_t/(1-p_t)$, corresponding to the probability equals to ${OR}_0p_t/(1-p_t+{OR}_0p_t)$, under $H_1$ the odds of shock for *t* th observation is ${OR}_1p_t/(1-p_t)$, the corresponding probability is ${OR}_1p_t/(1-p_t+{OR}_1p_t)$. To monitor the shift of $p_t$ individually, the log-likelihood ratio score based on the probability density function of the ZIP distribution is:

9 $$\begin{aligned} W_{t}=\left\{ \begin{array}{l} \log \frac{1-p_{t}+O R_{1} p_{t} e^{-\uplambda _{t}}}{1-p_{t}+O R_{0} p_{t} e^{-\uplambda _{t}}} \frac{1-p_{t}+O R_{0} p_{t}}{1-p_{t}+O R_{1} p_{t}} , \quad X_{t}=0 \\ \log \frac{O R_{1}}{O R_{0}} \frac{1-p_{t}+O R_{0} p_{t}}{1-p_{t}+O R_{1} p_{t}},\quad X_{t}>0 \end{array}\right. \end{aligned}$$

Combined with the Eq. (7), the score $W_t$ of *p*-CUSUM can be writen as:

10 $$\begin{aligned} W_{t}=\left\{ \begin{array}{l} \log \frac{1+O R_{1} e^{\varvec{\beta } \varvec{z}+k} e^{-e^{\varvec{\alpha }\varvec{x}+c}}}{{1+O R_{0} e^{\varvec{\beta } \varvec{z}+k}}e^{-e^{\varvec{\alpha }\varvec{x}+c}}} \frac{1+O R_{0} e^{\varvec{\beta } \varvec{z}+k}}{1+O R_{1} e^{\varvec{\beta } \varvec{z}+k}} , \quad X_{t}=0 \\ \log \frac{O R_{1}}{O R_{0}} \frac{1+O R_{0} e^{\varvec{\beta } \varvec{z}+k}}{1+O R_{1} e^{\varvec{\beta } \varvec{z}+k}},\quad X_{t}>0 \end{array}\right. \end{aligned}$$

Let ${RR}_0$ and $RR_1$ represent the relative risks under null and alternate hypotheses respectively. The mean number of cases for *t* th observation is $\uplambda _t$, Under $H_0$ the mean number is ${RR}_0\uplambda _t$, and under $H_1$ it is ${RR}_1\uplambda _t$. To monitor the shift of $\uplambda _t$ individually, the log-likelihood ratio score based on the probability density function of the ZIP distribution is:

11 $$\begin{aligned} W_{t}=\left\{ \begin{array}{l} \log \frac{1-p_{t}+p_{t} e^{-R R_{1} \uplambda _{t}}}{1-p_{t}+p_{t} e^{-R R_{0} \uplambda _{t}}}, \quad X_{t}=0 \\ X_{t} \log \frac{R R_{1}}{R R_{0}}+\left( R R_{0} \uplambda _{t}-R R_{1} \uplambda _{t}\right) ,\quad X_{t}>0 \end{array}\right. \end{aligned}$$

Substitute Eq. (7) into Eq. (11), the score $W_t$ can be:

12 $$\begin{aligned} W_{t}=\left\{ \begin{array}{l} \log \frac{1+e^{\varvec{\beta }\varvec{z}+k} e^{-R R_{1} e^{\varvec{\alpha }\varvec{x}+c}}}{1+e^{\varvec{\beta }\varvec{z}+k} e^{-R R_{0} e^{\varvec{\alpha }\varvec{x}+c}}}, \quad X_{t}=0 \\ X_{t} \log \frac{R R_{1}}{R R_{0}}+(R R_{0} -R R_{1}) e^{\varvec{\alpha }\varvec{x}+c},\quad X_{t}>0 \end{array}\right. \end{aligned}$$

To detect the shifts of the two parameters *p* and $\uplambda$ simultaneously, the score $W_t$ of the *t*-CUSUM can be defined as follow:

13 $$\begin{aligned} W_{t}=\left\{ \begin{array}{l} \log \frac{1-p_{t}+O R_{1} p_{t} e^{-R R_{1} \uplambda _{t}}}{1-p_{t}+O R_{0} p_{t} e^{-R R_{0} \uplambda _{t}}} \frac{1-p_{t}+O R_{0} p_{t}}{1-p_{t}+O R_{1} p_{t}},\quad X_{t}=0 \\ X_{t} \log \frac{R R_{1}}{R R_{0}}+\left( R R_{0} \uplambda _{t}-R R_{1} \uplambda _{t}\right) +\log \frac{O R_{1}}{O R_{0}} \frac{1-p_{t}+O R_{0} p_{t}}{1-p_{t}+O R_{1} p_{t}},\quad X_{t}>0 \end{array}\right. \end{aligned}$$

Substitute Eq. (7) into Eq. (13), the score $W_t$ can be written as:

14 $$\begin{aligned} W_{t}=\left\{ \begin{array}{l} \log \frac{1+O R_{1} e^{\varvec{\beta } \varvec{z}+k} e^{-R R_{1} e^{\varvec{\alpha }\varvec{x}+c}}}{{1+O R_{0} e^{\varvec{\beta } \varvec{z}+k}}e^{-R R_{0} e^{\varvec{\alpha }\varvec{x}+c}}} \frac{1+O R_{0} e^{\varvec{\beta } \varvec{z}+k}}{1+O R_{1} e^{\varvec{\beta } \varvec{z}+k}} , \quad X_{t}=0 \\ X_{t} \log \frac{R R_{1}}{R R_{0}}+(R R_{0} -R R_{1}) e^{\varvec{\alpha }\varvec{x}+c}+\log \frac{O R_{1}}{O R_{0}} \frac{1+O R_{0} e^{\varvec{\beta } \varvec{z}+k}}{1+O R_{1} e^{\varvec{\beta } \varvec{z}+k}},\quad X_{t}>0 \end{array}\right. \end{aligned}$$

Whenever the CUSUM statistics $C_t$ exceeds the control limit *h*, the control chart signals so that the practitioners can find the processes out of control. We use the average run length(ARL) to represent the performance of the control chart, it is defined as the average number of the observations before the CUSUM statistics first exceeds the control limit. We want the ARL under $H_0$, i.e. under the in-control condition, which called ARL$_0$, as large as possible, to achieve low rate of false alarm. And at the same time we want ARL$_1$, the ARL under $H_1$, as small as possible to detect the shift quickly.

## Results

### Simulation

In the simulation study, we use a variable $x_t$ as the impact factor of the *t* th observation. Correspondingly, we use one-dimension coefficient to generate the number of flu cases. We consider two different distribution of the impact factor $x_t$, respectively following N(0,1) and N(1,1). And we choose different coefficients for comparison. The values of the coefficients are respectively (a) $\beta$ = 0.5, $k = -1.386$, $\alpha$ = 0.5, *c* = 0 for $x_t\sim$ N(0,1); (b) $\beta$ = 0.5, $k = -1.386$, $\alpha$ = 0.5, *c* = 0 for $x_t\sim$ N(1,1); (c) $\beta = -0.5$, $k = -1.386$, $\alpha = -0.5$, *c* = 0 for $x_t\sim$N(1,1). In practice, the coefficients need to be estimated from the in-control samples by ZIP-regression but here we just assume they are known. To compare the risk-adjusted CUSUM with the unadjusted standard CUSUM, we consider three cases of parameter settings: (a) $p_0$ = 0.2, $\uplambda _0$ = 1.14; (b) $p_0$ = 0.3, $\uplambda _0$ = 1.87; (c) $p_0$ = 0.14, $\uplambda _0$ = 0.68. For better comparison the in-control parameters are chosen to be close to the mean of values of the parameters of the generated in-control data. In practice the values can be estimated from the in-control samples by MLE.

The pre-determined shift sizes of the parameter *p*-CUSUM based on the odds ratio(OR) are OR $\in \{1.5, 2, 4\}$, the shift sizes of the parameter $\uplambda _1$ based on the relative ratio(RR) are RR $\in \{1.5, 2\}$. For *t*-CUSUM the shift sizes are set as OR $\in \{1.5, 2\}$ for *p* and RR $\in \{1.5, 2\}$ to detect different parameter shifts. The out-of-control parameter value of *p* is $p_1=R_pp_0/(1+(R_p-1)p_0)$. The out-of-control parameter value of $\uplambda$ is $\uplambda _1=R_l\uplambda _0$. The shifts of the parameters may be simultaneously or individually. In practice, the pre-determined shift sizes indicate the out-of-control (i.e. “abnormal”) state. For example, the OR=1.5 in CUSUM means that the odds is expected to be 1.5 times as “normal” state. The CUSUM chart will achieve the best detection efficiency when the practical increase in odds is 50%. The shift size is usually based on the experience or the previous results to determine the out-of-control state in practice. In this study, we focus on the performance of the proposed chart for in-control state, because high false alarm rate for “normal” state may attenuate the applicability of the monitoring methods. The shift sizes may have little impact when the process is in-control. Three simple shift sizes are set to evaluate the in-control performance of the proposed method in different conditions.

Control limits of CUSUM control chart can be obtained from simulation or using the Markov chain method. In this paper, we obtain the control limits by 10000 replications simulation. Control limit is the threshold that is used to determine the state of process. The process could be specified as in-control (i.e. “normal”) when the upward CUSUM value is smaller than the control limit. The alarm is triggered and the process is concluded as out-of-control (i.e. significant deviation from “normal”) when the CUSUM value is above the such limit. ARL$_0$ is chosen to be 400 here. Control limits of the standard and risk-adjusted CUSUM control charts are provided in Tables 1, 2, 3 respectively for *p*-CUSUM, $\uplambda$-CUSUM and *t*-CUSUM.

**Table 1:** Control limits of the standard *p*-CUSUM chart and the risk-adjusted *p*-CUSUM control chart

	Case(a)		Case(b)		Case(c)	
	Standard CUSUM	Risk-adjusted CUSUM	Standard CUSUM	Risk-adjusted CUSUM	Standard CUSUM	Risk-adjusted CUSUM
OR = 1.5	1.751	1.7317	2.066	1.99	1.395	1.403
OR = 2	2.45	2.41	2.8	2.708	2.018	2.018
OR = 4	3.44	3.352	3.79	3.65	2.984	2.94

**Table 2:** Control limits of the standard $\uplambda$-CUSUM chart and the risk-adjusted $\uplambda$-CUSUM control chart

	Case(a)		Case(b)		Case(c)	
	Standard CUSUM	Risk-adjusted CUSUM	Standard CUSUM	Risk-adjusted CUSUM	Standard CUSUM	Risk-adjusted CUSUM
RR = 1.5	1.79	1.93	2.42	2.5012	1.301	1.398
RR = 2	2.3258	2.417	2.9821	2.925	1.7951	1.873

**Table 3:** Control limits of the standard *t*-CUSUM chart and the risk-adjusted *t*-CUSUM control chart

	Case(a)		Case(b)		Case(c)	
	Standard CUSUM	Risk-adjusted CUSUM	Standard CUSUM	Risk-adjusted CUSUM	Standard CUSUM	Risk-adjusted CUSUM
OR = 1.5 RR = 1.5	2.486	2.532	2.92	2.938	2.1088	2.113
OR = 1.5 RR = 2	2.793	2.839	3.2478	3.244	2.4303	2.4025
OR = 2 RR = 1.5	2.9535	2.939	3.2793	3.28	2.557	2.547
OR = 2 RR = 2	3.0789	3.1368	3.413	3.475	2.7535	2.75

Since the standard CUSUM control chart consider the parameters as the constants, there is a great possibility to generate false alarm, even though it can detect the shift faster than the adjusted chart, the high false alarm rate may reduce the credibility of the alarm. We do the simulation study to verify it. The comparison of the in-control performance among the standard CUSUM chart and the risk-adjusted CUSUM chart is provided in Tables 4, 5, 6, corresponding respectively to the *p*-CUSUM, $\uplambda$-CUSUM and *t*-CUSUM. The values in the Tables 4, 5, 6 are the average run length (ARL) of the CUSUM charts, which indicate the average number of observations required to signal. For all charts, the ARL of in-control process is fixed at 400, indicating that the alarm is mistakenly generated for about every 400 “normal” cases. The actual ARL under 400 suggests that the chart has higher false alarm rate when state is “normal”. The ARL closest to the in-control ARL 400  are  bolded in Tables 4, 5, 6.

**Table 4:** In-control ARL performance of the standard *p*-CUSUM chart and the risk-adjusted *p*-CUSUM control chart

	Case(a)		Case(b)		Case(c)	
	Standard CUSUM	Risk-adjusted CUSUM	Standard CUSUM	Risk-adjusted CUSUM	Standard CUSUM	Risk-adjusted CUSUM
OR = 1.5	266.6171	**399.5418**	442.7546	**399.9784**	277.2445	**401.4471**
OR = 2	275.8945	**397.2131**	433.6142	**400.0877**	279.4643	**397.6834**
OR = 4	295.9394	**399.7799**	431.744	**400.8284**	285.3585	**400.4745**

The ARL closest to the pre-determined in-control ARL 400 are bolded

**Table 5:** In-control ARL performance of the standard $\uplambda$-CUSUM chart and the risk-adjusted $\uplambda$-CUSUM control chart

	Case(a)		Case(b)		Case(c)	
	Standard CUSUM	Risk-adjusted CUSUM	Standard CUSUM	Risk-adjusted CUSUM	Standard CUSUM	Risk-adjusted CUSUM
RR = 1.5	100.2283	**398.9045**	70.7917	**405.22**	150.5684	**395.6469**
RR = 2	103.3361	**402.9761**	72.7272	**395.1574**	147.2813	**398.4174**

The ARL closest to the pre-determined in-control ARL 400 are bolded

**Table 6:** In-control ARL performance of the standard *t*-CUSUM chart and the risk-adjusted *t*-CUSUM control chart

	Case(a)		Case(b)		Case(c)	
	Standard CUSUM	Risk-adjusted CUSUM	Standard CUSUM	Risk-adjusted CUSUM	Standard CUSUM	Risk-adjusted CUSUM
OR = 1.5 RR = 1.5	115.6095	**400.8448**	83.9269	**404.663**	170.3881	**401.9765**
OR = 1.5 RR = 2	108.8625	**399.7592**	75.3776	**397.4291**	153.1147	**399.5158**
OR = 2 RR = 1.5	133.4295	**399.8585**	94.1814	**404.4315**	183.7638	**399.4048**
OR = 2 RR = 2	111.4156	**399.4888**	78.5196	**404.2784**	167.9807	**401.3558**

The ARL closest to the pre-determined in-control ARL 400 are bolded

From the table it is obvious that when in-control processes are monitored by standard CUSUM chart, the ARL is quite unstable, the ARL is much smaller than 400 except for the *p*-CUSUM under case(b), under this condition the ARL is much larger than 400. While our adjusted CUSUM chart can be stable at 400. Especially for the $\uplambda$-CUSUM and *t*-CUSUM, the ARL$_0$ of standard CUSUM is far from 400, which means that the standard CUSUM control chart generate false alarm frequently. The adjusted CUSUM control chart thus can be more accurate and credible.

In practice, when monitoring the influenza data, there is another important factor we should take into consideration—the seasonality. Previous studies suggested that the influenza activities data shows obvious seasonality. It is well known that winter is the peak season of influenza, however when construct the CUSUM chart, random sampling is used to obtain the control limit, if the seasonality is not considered, the control limit may be unstable, thus quantities of false alarm will be generated in the winter season, which highly reduces the accuracy of the prediction. Zhang et al. [26] demonstrated that eliminating seasonal effects can make the incidence trend more clear. So we do a simulation study assuming only the seasonality is considered as the impact factor when do the adjustment to show the effect clearly. Serfling [27] proposed a method of Fourier series with linear trend to monitor pneumonia-influenza data. The method is the combination of linear trend with a sine or cosine term describing seasonality. Thompson et al. [28] used a Poisson regression model to estimate influenza-associated deaths, in which seasonality is also described by Fourier series. In our paper, we use a cosine Fourier term to describe the seasonality.

We assume that the year we consider has 365 days and 4 seasons, in this simulation we consider seasonality as the only impact factor to adjust parameters *p* and $\uplambda$ to show the effect clearly, the regression is made using the Fourier series type approach, the coefficients are set to *b* = 0.5, *u* = -1.386, *c* = 0.5, *k* = 0.2. Let t represents the day of the year, the ZIP regression model can be written as:

15 $$\begin{aligned} \begin{aligned} \log (\uplambda )&=c [cos(2\pi t/4)]+k \\ \log i t(p)=\log \left( \frac{p}{1-p}\right)&=b [cos(2\pi t/4)]+u \end{aligned} \end{aligned}$$

We use shift sizes OR = 1.5 for *p*-CUSUM, RR = 1.5 for $\uplambda$-CUSUM and OR = 1.5, RR = 1.5 for *t*-CUSUM to monitor the changes. The comparison among the standard and adjusted CUSUM chart is shown in Table 7. From the table it is obvious that the ARL$_0$ of standard CUSUM is much smaller than the pre-determined value 400, indicating that it may be unstable and have high false alarm rate when monitoring the seasonal data. And the adjusted CUSUM has stable in-control performance, as we expected. That’s the reason why we choose risk-adjusted CUSUM control chart instead of standard CUSUM control chart to monitor the influenza data.

**Table 7:** Comparison of the in-control performance among the standard and risk-adjusted CUSUM chart to monitor seasonality

	*p*-CUSUM	$\uplambda$-CUSUM	*t*-CUSUM
Standard CUSUM	100.2283	196.8143	124.6451
Risk-adjusted CUSUM	**402.9923**	**400.5468**	**404.3959**

The ARL closest to the pre-determined in-control ARL 400 are bolded

To demonstrate the good performance of the proposed risk-adjusted CUSUM charts comparing to the widely used influenza surveillance monitoring method, we choose the time series method proposed by Cowling et al. [2] to monitor our simulation data under in-control condition. We use the same parameters setting as they did and use the same coefficients respectively to compare the performance of the time-series method and the proposed method. And we set the value of the parameter $m_0$ based on the mean of the underlying system process at the beginning. The ARL performance of the time-series method to generate alerts under in-control conditions are shown in Table 8. From the results it is obvious that the time-series method generates false alarm quite earlier than we expect, which demonstrates the instability of the traditional influenza surveillance monitoring method. By contrast, the proposed risk-adjusted CUSUM control charts can be more stable and accurate, and generate less false alarm.

**Table 8:** The ARL performance of the time-series influenza surveillance monitoring method under in-control condition

	Case(a)	Case(b)	Case(c)
Time series method	32.4394	29.9562	47.9432

### Application

In this section, the Hong Kong influenza data is used to demonstrate the application of the risk-adjusted CUSUM control chart. The data of numbers of Hong Kong influenza cases is collected from Prince of Wales Hospital. And the daily climate data comes from the Hong Kong Observatory(HKO). There are 12 factors that may have an impact on the influenza activities collected in total. Several researches studied the parameters that may have an effect on the influenza activities, including environment and social factors [15–18, 20]. The recent study about association between the climate factors and the activities of influenza A and B respectively by Chong et al. [19]. found that temperature has a decisive influence on influenza activities in no matter what climate regions, humidity is also an important factor. In the regression model they made, daily precipitation amount, wind speed and public holidays are all the factors they included. For Hong Kong data, temperature has the most contribution to the type A influenza activities, then wind speed, relative humidity and precipitation, public holiday seems no significant impact on it, as for type B influenza activities, temperature is still the most important, second is relative humidity, then wind speed and precipitation, public holiday has little impact. From their research it is obvious that the two types of flu cases should be considered separately. Based on these previous researches and the significance of the explanatory variables, we choose the appropriate factors with significant impact on the parameters as the regression factors, while seasonality is also considered here.

**Fig. 1:**
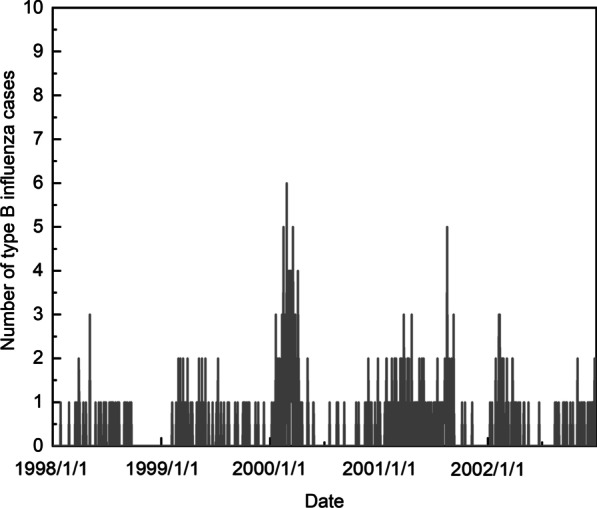
The number of type B influenza data cases during 1998–2002

To verify the in-control performance of our proposed method on influenza surveillance data with excess zeroes and low mean, we choose the type B influenza daily surveillance data of 1998–2001 as the in-control data to construct the control charts and the charts are used to monitor the type B influenza data of 2002. Daily data of numbers of the type B influenza cases from 1998 to 2002 is shown in Fig. 1. The number of type B influenza cases of 1998–2002 is averagely 1–2 per day in Fig. 1, indicating that the “normal” state may have this pattern. The increase of the number to a high level (e.g. 10 in later stage) may suggest the “abnormal” state and the alarm could be necessary. We choose precipitation, nitrogen dioxide, sulfur dioxide and the seasonal term as the impact factors on probability *p* and air temperature, humidity, precipitation, and Ozone as the impact factors on Poisson parameter $\uplambda$, based on the significance and the previous studies. The coefficients are obtained by ZIP regression from the 1998–2001 influenza data. Assuming that both parameters may shift, we use the t-CUSUM to monitor the 2002 influenza data and the pre-determined shift-size is $p_1=1.5p_0/(1+(1.5-1)p_0)$, $\uplambda _1$ = 1.5$\uplambda _0$. ARL$_0$ is determined to be 2000 here to obtain the control limit, which ensures there would be little false alarm generated in a in-control year. The time series method is also applied here to monitor this influenza surveillance data. From the data we can see that the mean of the underlying system process at the beginning can be set to 1, to compare with the CUSUM charts we set the confidence as 99.975%.

**Fig. 2:**
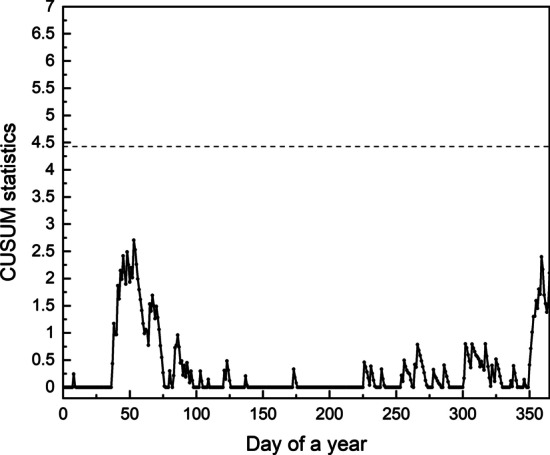
Risk-adjusted CUSUM chart for 2002 year influenza monitoring

**Fig. 3:**
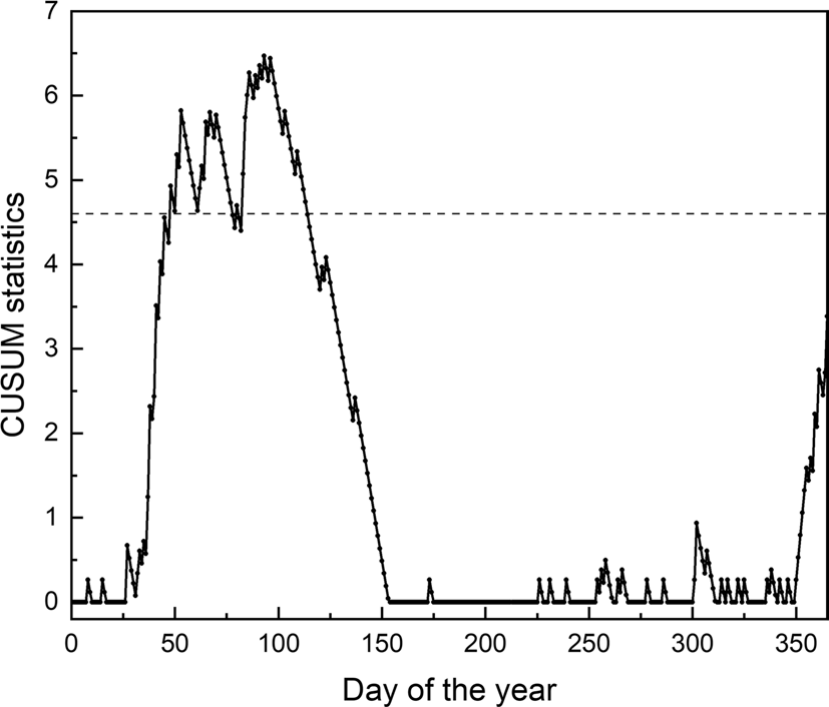
Standard CUSUM chart for 2002 year influenza monitoring

**Fig. 4:**
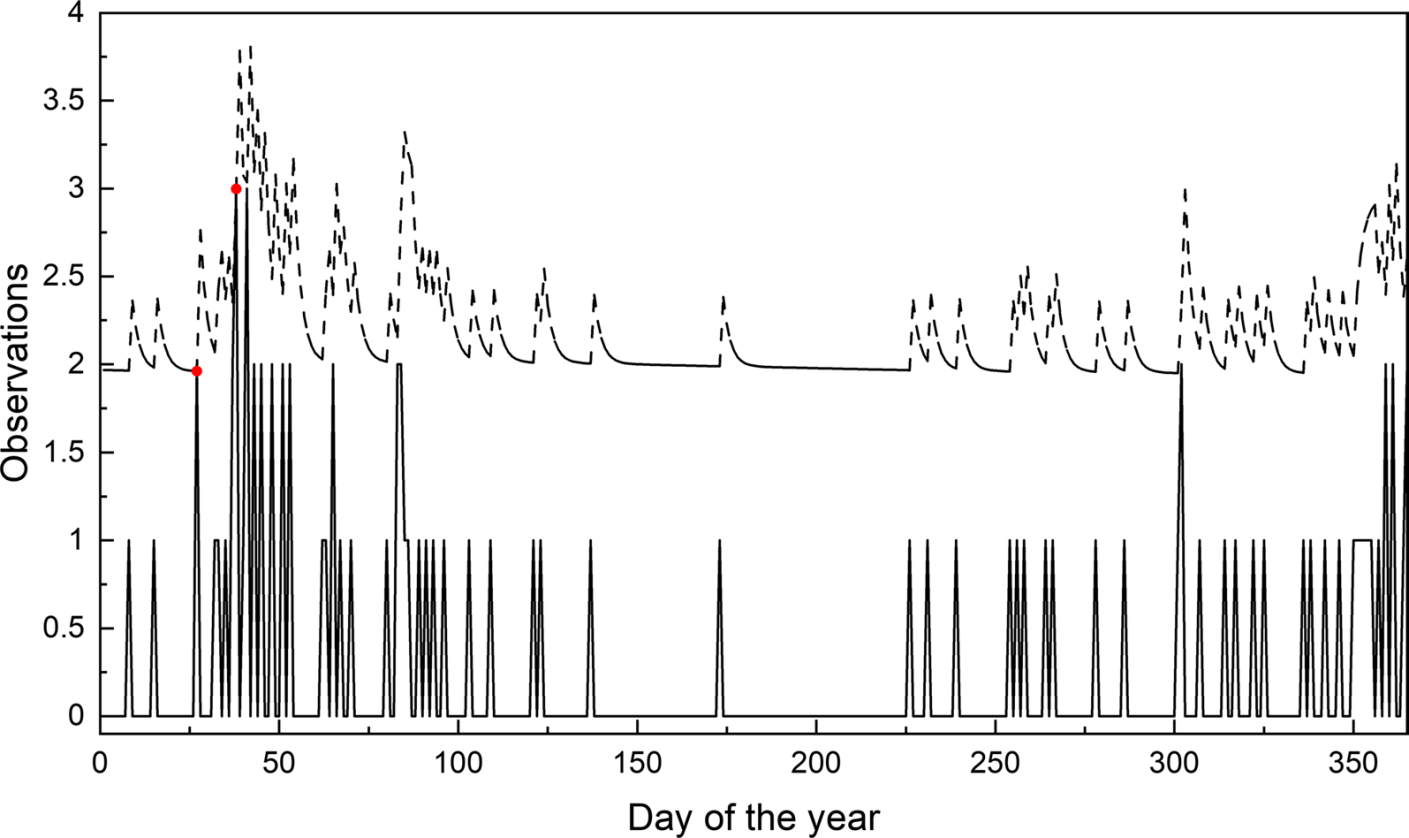
Time series method monitoring chart for 2002 year influenza monitoring

As Fig. 1 shows, the number of influenza cases of 2002 is in normal, there is no need to raise an alarm this year. We use the adjusted CUSUM chart, standard CUSUM chart and time series method respectively to monitor the 2002 data. The risk-adjusted CUSUM chart is shown in Fig. 2, the standard CUSUM chart in Fig. 3 and the chart of the time series method can be seen in Fig. 4. In CUSUM charts, the solid line represents the CUSUM statistics and the dashed line represents the control limit. In time series method chart, the solid line represents the observations and the dashed line represents the corresponding forecast limits. As Fig. 3 shows, the standard CUSUM signals frequently from the 48th day of 2002, it is obviously unreasonable. The red dots in Fig. 4 represent the time when the chart alerts. From Fig. 4 we can find that the chart generates false alarms on the 27th and 38th day of 2002, which shows the instability of the method. As the parameter $m_0$ and $C_0$ are determined based on the input data, the method depends on the experience of the practitioner a lot, which may cause some mistakes or reduce the performance. While the chart proposed in this paper will not signal, as Fig. 2 shows, demonstrating the low false alarm rate of our chart.

## Discussion

The simulation results show that the adjusted CUSUM charts have stable ARL at 400 as we expected while the standard charts far from 400. The application suggests that the proposed adjusted CUSUM charts can also perform well on real data. From the results of the simulation study and the application on a hospital, we can find that the adjusted ZIP-CUSUM control chart is more stable than the standard ZIP-CUSUM control chart when the process is in-control. Without adjusting the impact of risk factors, the standard CUSUM chart would generate higher false alarm rate when there is no change. The proposed adjusted ZIP-CUSUM chart can monitor the influenza with expected in-control ARL. It should be noted that the pre-determined shift sizes of the CUSUM charts have an influence on the performance of the charts, but it is not easy to predict the real shift size of a out-of-control process, which may reduce the efficiency the CUSUM control charts. Therefore, it is meaningful to consider a monitoring method in future study in which the value of alternative hypothesis is not necessary.

## Conclusion

In this paper, we propose a risk-adjusted CUSUM control chart to monitor the zero-inflated Poisson processes. This chart uses the ZIP regression to adjust the impact of risk factors when implementing influenza monitoring. The new adjusted CUSUM control charts can be more accurate and stable. Comparing with the standard CUSUM charts without risk adjustment, the adjusted CUSUM charts can achieve the designed ARL$_0$, i.e. the standard ZIP CUSUM chart has unexpected higher false alarm rate. Therefore, the alert triggered by the proposed adjusted chart may be more credible and then may help to reduce the waste of medical resources that caused by frequent false alarm. Thus the adjusted CUSUM chart can be an useful tool in monitoring the influenza pandemic.

## Data Availability

The datasets generated and analyzed during the current study are available upon reasonable request.

## Abbreviations

ZIP: Zero-inflated Poisson
SPC: Statistical process control
UCL: Upper control limit
GZIP: Generalized zero-inflated Poisson
CUSUM: Cumulative sum
EWMA: Exponentially weighted moving average
CRL: Conforming run length
ZTP: Zero truncated Poisson
AEWMA: Adaptive exponentially weighted moving average
RMI: Relative mean index
GWMA: Generally weighted moving average
DEWMA: Double exponentially weighted moving average
LST: Land Surface Temperature
SARIMA: Seasonal autoregressive integrated moving average
pdf: Probability density function
MLE: Maximum likelihood estimation
OR: Odds ratio
RR: Relative ratio
ARL: Average run length
HKO: Hong Kong Observatory
